# Constant performance in balance and proprioception tests across the menstrual cycle – a pilot study in well trained female ice hockey players on hormonal contraception

**DOI:** 10.1002/hsr2.18

**Published:** 2017-11-24

**Authors:** Kirsten Legerlotz, Marie Elena Bey, Susann Götz, Nikolai Böhlke

**Affiliations:** ^1^ Department of Training and Movement Sciences Humboldt University Berlin Germany; ^2^ Division of Training and Movement Science University of Potsdam Potsdam Germany; ^3^ Olympic Training Centre Berlin Berlin Germany

**Keywords:** female athlete, injury risk, balance, body composition, biomechanics, hormones

## Abstract

**Introduction:**

It has yet to be explained why female athletes appear to suffer more often from non‐contact ligament injuries during the first half of the menstrual cycle. Fluctuations in balance, caused by impaired proprioception due to increased fluid retention, may be relevant factors contributing to this anomaly in distribution. The aim of this study was therefore to uncover relationships between dynamic stability, proprioception and fluid retention in association with the menstrual cycle as a possible explanation for the observed changes in injury rates.

**Methods:**

Nine healthy, female, well trained ice hockey players on hormonal contraception with regular cycles were tested at least twelve times during one menstrual cycle. Bioimpedance analysis was applied to investigate body composition and fluid distribution. A joint position sense test was performed to characterize changes in proprioception, while unexpected perturbations on a balance board were used to obtain measures for dynamic postural control.

**Results:**

No distinct changes in proprioception, dynamic stability and fluid retention were identified across the menstrual cycle in this population. Weak correlations were found between parameters for dynamic stability and proprioception.

**Conclusion:**

Variations in proprioception, dynamic stability and fluid retention seem unlikely to be major contributors to injury risk peaks, at least in this population of healthy trained athletes using hormonal contraception.

## 1 INTRODUCTION

The general physiological function and associated hormonal changes during the female menstrual cycle are known for decades and in medical text books well documented.^1^, ^2^ However, its specific effects in the context of physical activity and sports performance are still debated.^3^, ^4^, ^5^, ^6^ Some studies have shown small effects on athletic performance, as e.g. a reduction in VO_2max_ in the luteal compared to the follicular phase,^6^ or a higher heart rate during sub‐maximal exercise in the luteal compared to the follicular phase,^7^ while others found no difference.^5^, ^8^ Furthermore, the trainability appears to change during the menstrual cycle, with strength training being more effective, resulting in a higher increase in muscle diameter and maximum strength, when performed in the follicular compared to the luteal phase.^9^ This has been explained with the specific hormonal status in the follicular phase.^9^

Female sex hormones have, amongst other factors, been suspected to negatively affect structure and mechanical properties of ligaments, making them more prone to injury,^10^ since the incidence of anterior cruciate ligament (ACL) injuries is also in general higher in women than in men.^11^ This is why numerous studies have tried to link a specific phase of the menstrual cycle associated with specific levels of estrogen and progesterone to the ACL injury risk.^12^, ^13^, ^14^, ^15^, ^16^, ^17^, ^18^ However, results from studies differ widely, and even reviews vary in their conclusions.^19^, ^20^, ^21^ While one review stated that definite conclusions are not warranted regarding the association between menstrual cycle and ACL injury risk,^21^ another review identified a predisposition to injury in the pre‐ovulatory phase^20^ and yet another one suggested that the injury risk was increased during the ovulatory phase.^19^

Serum estradiol concentrations peak in the ovulatory phase, which is prevented by using oral contraception, leading to constant level of serum estradiol concentrations.^22^ However, despite a more constant hormonal profile when using hormonal contraception fluctuations in ACL injury prevalence appear to persist,^23^ although the overall risk has been reported to decrease when using oral contraceptives.^24^, ^25^

While to our knowledge a reliable explanation for the mechanisms underpinning the observed fluctuations in injury risk with and without the use of hormonal contraception along the menstrual cycle has not been given yet, several factors have been discussed. For example, significant associations between menstrual cycle phase and knee laxity have been observed, with increases during the ovulatory or luteal phases of the cycle in women not using hormonal contraception,^26^ suggesting a change in ligament compliance. However, this could at least be partly due to the ovulation associated rise in body temperature when not using hormonal contraception rather than to changes of the intrinsic properties of the ligament, since menstrual cycle associated differences in muscle and tendon flexibility disappear when the tested leg is warmed to 38 °C.^27^ In contrast, measures for knee laxity such as ACL elasticity, force to flex the knee and knee flexion‐extension hysteresis have been shown to be constant in women using oral contraceptives, while differences between contraceptive users and non‐users in force to flex the knee and knee flexion‐extension hysteresis disappear after warming the leg to 38°.^22^

More importantly, the adaptation of tendons to training is usually only measurable after a training duration of at least two months.^28^, ^29^ This suggests that significant changes of the morphological properties of this type of tissue seem rather unlikely to occur within a couple of days during a specific phase of the cycle. Thus, changes in the morphological properties of tendons and ligaments along the female cycle due to the changes in the hormonal milieu are unlikely to cause the observed variations in injury rates.

Another factor which could change more rapidly while affecting injury risk is postural control. This has been shown to change during the menstrual cycle, with lateral sway being greater just before and after menses (day 25 and 5 of the cycle) in cycles without using hormonal contraception.^30^ Fluctuations in postural control also occur in women using hormonal contraception.^31^ In fact one study even suggests that postural stability is reduced in contraceptive users compared to non‐users.^32^ The mechanism behind this change in postural control is also unclear. However, it might be related to a change in proprioception, as a decrease in knee joint position sense has been reported to occur at the same time, at menses.^33^, ^34^ Again, this change in proprioception might not be directly linked to specific hormone levels, but to accessory phenomenon such as e.g. menses associated fluid retention, affecting sensory feedback from the mechanoreceptors in the muscle. Whilst there was no significant association between estradiol or progesterone levels, self‐reported fluid retention scores also peaked at the onset of menses.^35^

The aim of this study was therefore to identify possible relationships between postural control, proprioception and fluid retention and to observe variations along the menstrual cycle in female athletes on hormonal contraception. If these relationships can be identified, they might provide a better explanation for the observed changes in injury rates along the menstrual cycle. In turn, this might help female athletes and their coaches to address these issues by adjusting their training accordingly.

As subtle changes in performance are both more relevant to as well as easier to identify in elite athletes than in the normal population, the study was conducted with a homogenous group of well‐trained female ice hockey players on hormonal contraception. We hypothesized, that total body water would increase around menses, being accompanied by a reduction in joint position sense and measures of dynamic postural control.

## 2 METHODS

The study was performed with nine healthy female well‐trained (national level) ice hockey players (age: 22 ± 7 years; height: 168 ± 5 cm; weight: 61 ± 6 kg). They were tested during the general preparation period between May and July, while training 10 ± 2 hours per week. The athletes continued with their normal training regime during the study. All players were using hormonal contraception containing a combination of two active substances, estrogen and progesterone. All contraceptives contained Ethinylestradiol as first active substance. When orally taken the concentration of Ethinylestradiol was 0.02‐0.03 mg per pill (eight players) and 2.7 mg per vaginal ring (one player). The second active substance was Levonorgestrel (0.1‐0.15 mg per pill; 5 players), Chlormadinone (2 mg per pill, two players), Dienogest (2 mg per pill, one player) or Etonogestrel (11.7 mg per vaginal ring, one player).

Local institutional review board (Humboldt University Berlin, Faculty of Humanities and Social Sciences) approval was obtained. Written, informed consent was given, and the study was performed in compliance with the Declaration of Helsinki.

After two pre sessions, which were used to familiarize the subjects with the dynamic stability test to minimize training effects, the subjects were tested twelve to fourteen times during one full menstrual cycle for bioimpedance measures, dynamic stability and sense of joint position. The participants entered the study at a random point in their menstrual cycle, to exclude sequence effects. The first day of the last menses, the next menses and if necessary the second next menses were recorded. The test days were retrospectively allocated to a day in the menstrual cycle, ranging from day 1 as the onset of menses to day 28 as the day before onset of menses.

### 2.1 Bioimpedance measurement

For bioimpedance measurements the subjects stood bare feet and lightly dressed on the scale (InBody 720, Biospace Co., Korea). The subjects adopted an upright posture, with each foot being placed on two foot electrodes (heel and forefoot), each hand being in contact with two hand electrodes (fingers and thumb) and the arms held in an ~15° angle to the upper body. The bioelectrical impedance analysis was performed according to the manufacturer's instructions. Briefly, 30 impedance measurements per subject were performed using 6 different frequencies, from which segmental resistance was calculated. The following output parameters are reported as absolute values: body mass [BM, kg], skeletal muscle mass [SMM, kg], fat mass [FM, kg], total body water [TBW, l], extracellular water [ECW, l] and intracellular water [ICW, l]. In terms of accuracy of the eight‐point tactile‐electrode impedance method, the coefficient of variation for segmental resistance has been reported to be ≤ 2.8 %, calculated from three measurements performed on five consecutive days in 50 men and women.^36^ Measuring six women five times within one day the coefficients of variation have been calculated to be 0.1 ± 0.2% for BM, 0.4 ± 0.2% for SMM, 1.0 ± 0.4% for TBW, 0.3 ± 0.2% for ICW and 0.4 ± 0.2% for ECW.

### 2.2 Proprioception

**Figure 1:**
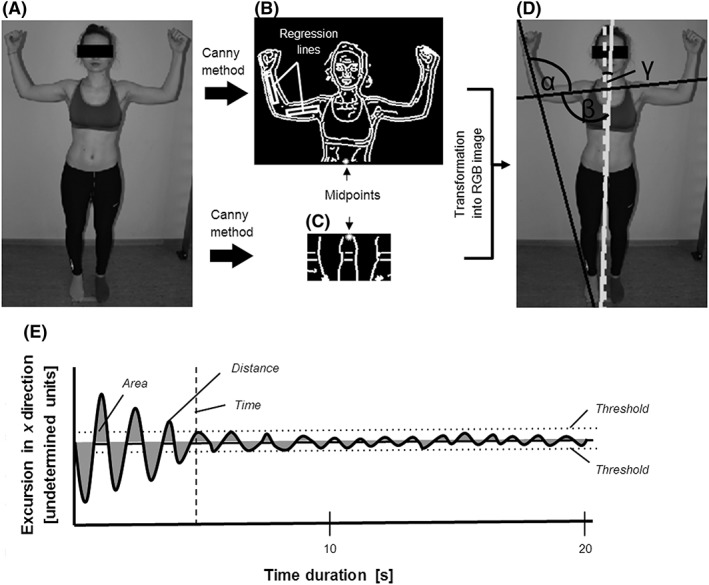
Depiction of data analysis: (A‐D) Quantitative objective image analysis of the JPS test & (E) analysis of dynamic postural control test. The original RGB image (A) is converted to a grayscale image of the upper (B) and lower body (C), identifying the edges of the subject using the Canny method. Regression lines within the arm segments are identified, describing their orientation. To generate the longitudinal body axis, the subject's center at waist and thigh height is identified and marked with a star. Thereafter the image is converted back to RGB image (D), showing the longitudinal body axis (white continuous line), the gravitational axis (white dashed line) and the orientation lines of the forearm and upper arm (black lines). The line orientation is used for the angle assessment of α, β, γ. (E) Time dependent data for platform movements in the *x* axis recorded during the dynamic postural control test. Derived parameters (time, area, distance and threshold) are marked

In order to obtain a quantifiable measure for proprioception, a joint position sense (JPS) test was performed, which records the awareness of the joints static location in space.^37^ A JPS test for the upper limb was developed, allowing marker‐less assessment of three angles by image recorded angulation. The subjects were asked to adopt the following target position with closed eyes: standing straight (0° deviation from gravitational axis), arms held at the side with a 90° angle between upper body and upper arm, and 90° angle between forearm and upper arm (Figure 1, A‐D). A digital image of the subject's frontal plane was taken under standardized conditions, with fixed positions of subject and camera.

Quantitative objective image analysis was performed using Matlab (R2015a, 64 Bit, The Mathworks, Natick, USA), to obtain the angles between forearm and upper arm (α), upper arm and longitudinal body axis (β) and longitudinal body axis and gravitational axis (γ). For image analysis the subject was isolated from the background by converting the original red‐green‐blue (RGB) image (Figure 1A) to grayscale. The edges of the subject were found using the Canny method (Figure 1B). The longitudinal body axis was determined by two points, the midpoint of the upper part of the body in waist height and the midpoint between both thighs close to the knees. The orientations of the arm segments were calculated by regression lines out of the midpoints between the outer edges of the forearm and the upper arm. Left and right half of the body were analyzed separately.

### 2.3 Dynamic postural control

In order to quantify dynamic postural control, the balance platform Posturomed (Haider Bioswing GmBH, Germany) was used. The Posturomed is widely applied as both training device^38^ and diagnostic tool.^39^ A good inter‐ and intra‐day reliability has been reported for the dynamic balance test on the Posturomed with intra‐class correlation coefficients ranging between 0.713 and 0.970.^40^ This vertically suspended, unstable balance platform, which is mounted to eight springs and has been described in detail elsewhere,^41^ was equipped with a lever‐based provocation unit allowing unexpected horizontal perturbations to be applied in a standardized manner. The Posturomed was further fitted with the associated accelerometer to record oscillations, using the provided software (MicroSwing 5.0, Haider Bioswing GmBH, Germany). For the measurements, the subjects were asked to stand bare feet on one leg in the center of the platform, with the knee of the supporting leg slightly bend, the thigh of the free leg being parallel to the supporting leg, and the hands placed on the hips, looking straight ahead. Two tests per leg were executed with the perturbations occurring once from medial and once from lateral, and the mean was calculated for each leg. The unexpected perturbation was triggered randomly within the first 5 seconds of each test. After perturbation the subjects were asked to regain balance and to stop the platform from oscillating as quickly as possible without using their arms. The subjects then continued to stand steadily on the platform until the end of the test period 20 seconds from perturbation. Time dependent data were recorded for platform movements in the *x* and *y* axis at a frequency of 50 Hz, analyzed with Matlab (R2015a, 64 Bit), from which four parameters were derived: 1) threshold, defined as maximum excursion in *x* direction during the regained balance phase [undetermined units], was determined individually for each subject within a time period of 7 seconds to the end of the test period. This threshold represents postural sway during balance; 2) time, defined as the time passed between the start of perturbation and reaching the threshold value in *x* direction [s]; 3) distance, defined as the length of the curve between the perturbation and the threshold in *x* or *y* direction [undetermined units] and 4) area, defined as the integral between the perturbation and the threshold in *x* or *y* direction [undetermined units] (Figure 1E).

### 2.4 Statistical analysis

A linear mixed‐effects model (LMM) was applied to detect statistically significant changes in the investigated parameters over the course of the menstrual cycle using Matlab (R2016a, 64 Bit, The Mathworks, Natick, USA). The LMM considers fixed effects (β_k_) describing population parameters, and random effects (b_ik_) which are associated with intrasubject differences over time.^42^ A biquadratic LMM which also includes a constant, linear, squared and cubic term was created using the function *fitlmematrix*. Taking into account the small sample size the function *REML* (restricted maximum likelihood method) was used to estimate the variance components. The model coefficients were tested combined using the hypothesis test *coefTest* with H_0_: β_1_ = β_2_ = β_3_ = β_4_ = 0. If the coefficients β_k_, k ϵ {1,…,4} are equal to zero the model is reduced to the constant term. The reduction to the constant term indicates an even distribution of the data with no time dependent effect.

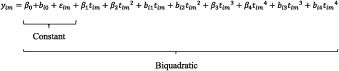

i: index for participant, i ϵ {1,…,9}
m: index for total number of measurements
t_im_: day of measurement, t ϵ {1,…,28}
β_k_: parameter for constant, linear, squared, cubic and biquadratic term, k ϵ {0,…,4}
b_ik_: individual parameter for linear, squared, cubic and biquadratic term, k ϵ {0,…,4}
ε_im_: residual

To establish relationships between parameters describing postural control, proprioception and body water independent of the menstrual phase, the Pearson correlation coefficient was calculated. For all statistical tests, significance was established at *P* ≤ 0.05.

## 3 RESULTS

### 3.1 Body composition

**Figure 2:**
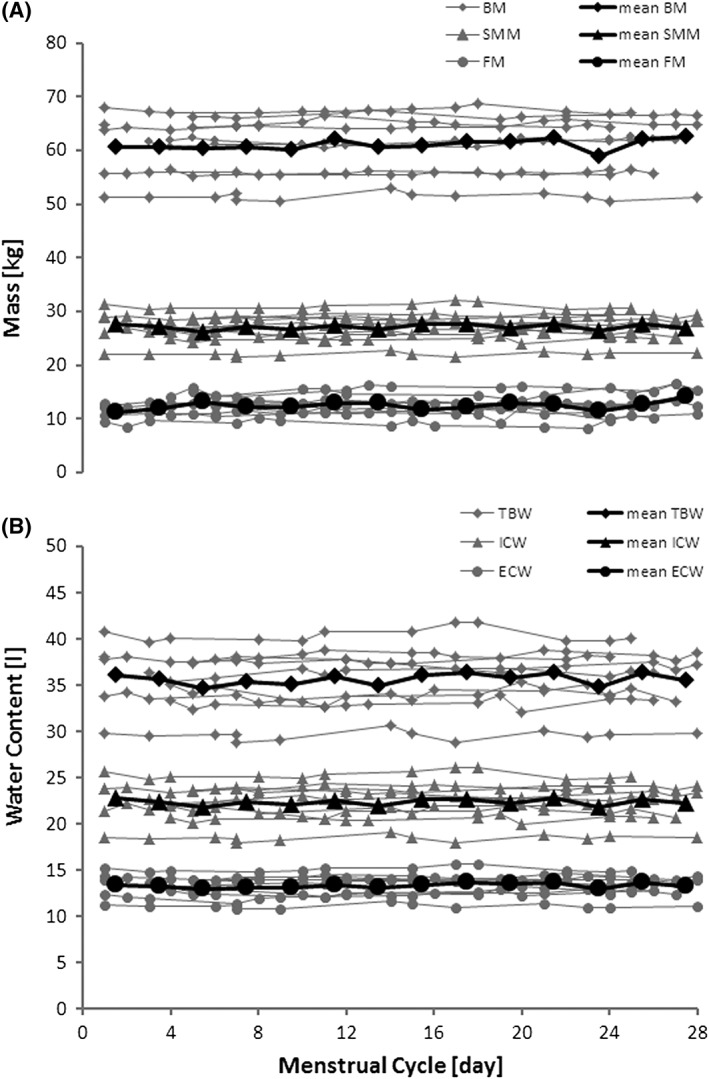
Body composition and water distribution along the menstrual cycle. (A) The body composition is represented by body mass (BM), skeletal muscle mass (SMM) and fat mass (FM); (B) Water distribution is described by total body water (TBW), extracellular water (ECW) and intracellular water (ICW). Grey symbols represent individual values. Black symbols represent mean values over all subjects within two days of the menstrual cycle

The athletes gained on average 0.37 kg skeletal muscle mass and lost 0.31 kg body fat during the test period, while their mean body mass, skeletal muscle mass and fat mass did not significantly change across the menstrual cycle (BM: P=0.44; SMM: P=0.66; FM: P=0.46; Figure 2A). Over all athletes, the mean deviation from the individual mean body mass was 0.7 ± 0.5 %, with a maximum individual deviation of 2.9 % or 1.5 kg, the mean deviation from individual mean fat mass was 3.8 ± 3.1 %, and the mean deviation from individual mean skeletal muscle mass was 1.3 ± 1.0 %. The mean relative skeletal muscle mass and mean relative fat mass were 44.4 ± 4.4 % and 20.3 ± 3.2 % relative to total body mass.

Regarding water distribution along the menstrual cycle the variables total body water and intracellular water did not vary (TBW: P=0.13; ICW: P=0.75), with the mean deviation from individual total body water being 1.2 ± 0.9 %, and the mean deviation from individual intracellular water being 1.2 ± 1.0 %. Significant changes were detected for extracellular water (P=0.01; Figure 2B) with the mean deviation from individual extracellular body water being 1.5 ± 1.3 %. Assessing the fixed effects coefficients β_k_, k ϵ {0,…,4} separately a tendency (P=0.09) for a linear trend was observed, with an incline of 0.01 l/day, while the constant model was still the best fit to describe the data (P=0.001).

### 3.2 Proprioception

**Figure 3:**
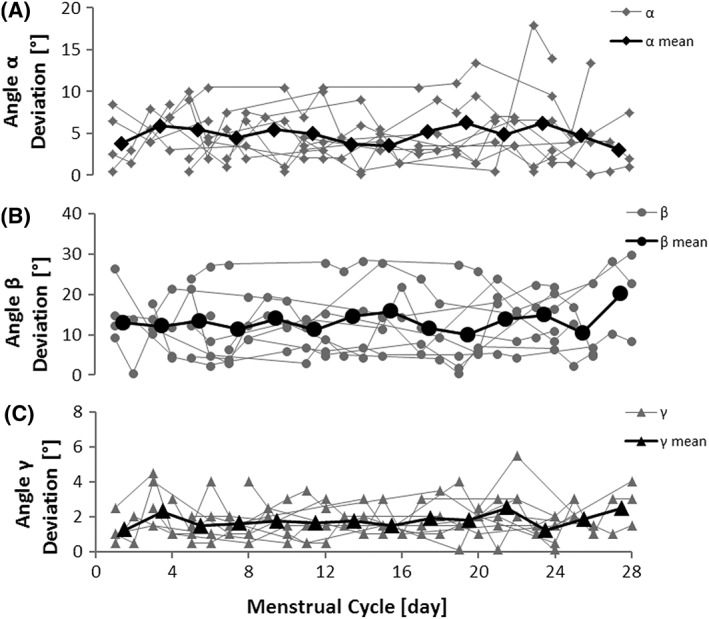
JPS test results along the menstrual cycle. Deviation from the anticipated target joint position at the JPS test for (A) the angle between forearm and upper arm (angle α); (B) between upper arm and longitudinal body axis (angle β); and (C) between longitudinal body axis and gravitational axis (angle γ). Grey symbols represent individual values. Black symbols represent mean values over all subjects within two days of the menstrual cycle

The deviation from the anticipated target joint position was largest for the angle between upper arm and longitudinal body axis (β) with a mean deviation of 13.0 ± 6.1°, followed by the angle between forearm and upper arm (α) with a mean deviation of 4.9 ± 1.9°, and the angle between longitudinal body axis and gravitational axis (γ) with a mean deviation of 1.8 ± 0.3°. None of the angles showed any significant variation during the menstrual cycle (α: P=0.81; β: P=0.52; γ: P=0.88; Figure 3).

### 3.3 Dynamic postural control

**Figure 4:**
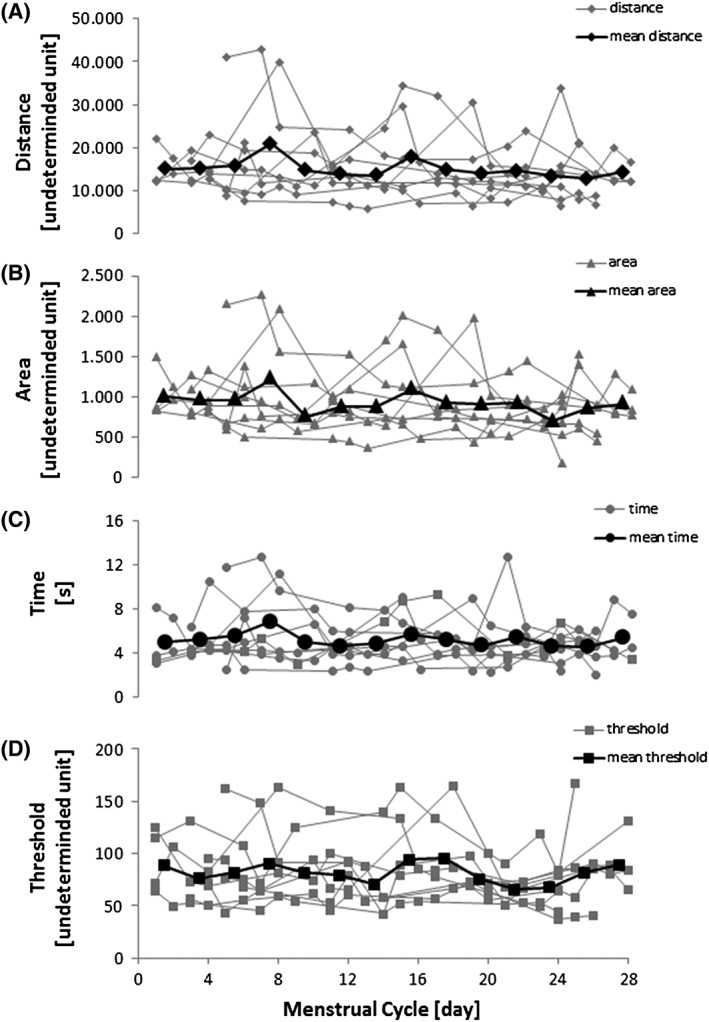
Quantification of dynamic stability along the menstrual cycle by using unexpected perturbations on a balance platform. Analysed parameters were: A) distance, B) area, C) time and D) threshold. Grey symbols represent individual values. Black symbols represent mean values over all subjects within two days of the menstrual cycle

Since there were no significant differences between the dominant and the non‐dominant leg for all measured parameters, the mean of both legs was calculated. The mean values for distance, time, area and threshold during regained balance did not vary significantly along the menstrual cycle (area: P=0.46; distance: P=0.51; threshold: P=0.46; time: P=0.35; Figure 4).

### 3.4 Relationships between postural control, proprioception and body water

No correlations were detected between body water and the parameters for proprioception or dynamic postural control. In terms of postural control and proprioception, weak but significant correlations were found between the parameter time and the angle β (*r*=‐0.25; P=0.012), and between the parameter threshold and the angle γ (*r*=0.22; P= 0.023).

## 4 DISCUSSION

In contrast to our hypothesis, no significant variations in body water, proprioception or dynamic stability were detected along the hormonally controlled menstrual cycle. Body water, proprioception and dynamic stability do not appear to be in close relationship to each other. Although two significant correlations were detected, the strength of these were weak. This suggests that a variety of other variables affects this relationship.

Fluid retention is one of the most common side effects linked to the menstrual cycle, with edema being localized not only in the breasts but also in upper and lower limbs.^43^ Since it is only possible to measure the water content of different parts of the human body indirectly, several techniques have been applied to assess it using volume^44^ and circumference^43^ measurements, bioelectrical impedance analysis^45^ or self‐reported rated impressions.^35^ It is a common finding that values increase during the second half of the cycle,^43^, ^44^, ^45^ peaking at the onset of menses,^35^ while the exact amount of the additional fluid is difficult to specify. For the breast an additional 100 ml have been measured^44^ and ~500 ml for total body water,^45^ with self‐reported fluid retention peaking at 0.9 on a 0 (none) ‐ 4 (very intense) scale.^35^ First, this indicates that the effect might be small, and could thus be missed in smaller populations. Secondly, fluid retention can be reduced^44^ or completely abolished^45^ through oral contraceptive intake. In our study a linear trend for an increase in extra‐cellular water along the menstrual cycle was detected. If this trend was real it might not be physiologically relevant as the incline was just 10 ml/day. Since the participants in our study were on hormonal contraception it is likely that at least some of them were not affected by fluid retention at all. Furthermore, exercise has been shown to reduce edema,^46^ thus the training in itself could have led to a reduction in fluid retention, thereby overriding the effects of the menstrual cycle. This could especially be the case for female athletes following a highly systematic and frequent training routine – as it was the case for the participants in our study. While the manufacturer of the bioimpedance scale recommends to measure women outside of menses, the remarkably constant body composition of our participants suggests that in a population such as ours muscle and fat mass can be reliably measured independent of the day in the menstrual cycle. Except for the individual variation in fat mass, which was with 3.8 % slightly higher than in the other parameters, mean individual variations were between 0.7 % and 1.5 %, thereby ranging above the intra‐day coefficient of variation calculated by ourselves and below the reported inter‐day coefficient of variation reported to be 2.8 %.^36^ These 2.8 % might however not entirely represent measurement error, as the intra‐day variation in that study was measured on five consecutive days, which is a timeframe in which physiological changes in body composition can occur.

Regarding proprioception contradictory results have been reported. While Fouladi et al.^33^ reported a decreased knee joint position sense accuracy at menses, Hertel et al.^47^ found no differences across the menstrual cycle. Both studies analyzed a similar sized group of young physically active women not using hormonal contraception, although with slight differences in the testing procedure. To our best knowledge, there are no studies on the effect of hormonal contraception on the joint position sense. The subjects in the above mentioned studies were regularly exercising or even competing, so a specific training regime or a competition prior to testing could have resulted in muscle fatigue, thereby influencing the joint position sense. The effect of a concomitant training on proprioception was reduced in our study, as the participants followed a stable training routine over the course of the data collection period that they were familiar with. In addition, the use of hormonal contraception in our study might also have stabilized the joint position sense.

Similar to proprioception, contradictory results have also been reported for postural stability. An increase in lateral sway just before and after menses compared to the rest of the cycle suggests postural stability to be affected by the menstrual cycle.^30^ However, several other studies could not detect any significant variations in postural stability across the menstrual cycle.^47^, ^48^, ^49^ Interestingly, this appears to change when women suffer from premenstrual symptoms. Postural sway is greater and kinaesthesia reduced in women with premenstrual symptoms compared to women without premenstrual symptoms.^49^ Postural sway is also greater in the luteal compared to the follicular and ovulatory phase in women suffering from premenstrual symptoms.^50^ Since aerobic exercise has been reported to reduce premenstrual symptoms, including fluid retention,^51^ it is conceivable that female athletes, as in our study, are in general less affected by menstrual cycle associated changes in postural control. Also oral contraceptives have been reported to reduce premenstrual symptoms,^52^ thereby possibly also reducing negative effects on postural control. However, if premenstrual symptoms still persist in spite of oral contraceptive intake the postural sway area in one‐legged stance has shown to be elevated during the active phase of treatment, while it remained constant in oral contraceptive users without premenstrual symptoms.^31^

Overall it was noticeable, that previous studies did not detect direct correlations between hormone levels and measures of postural control,^50^ proprioception^47^ or fluid retention.^35^ Similarly, tendon stiffness^53^ and knee laxity^47^ were not correlated to hormone levels. Thus it might be too simplistic to assume a direct relationship between those variables and menstrual cycle associated hormone levels. This might indicate that factors secondary to hormone levels or a combination of several factors are responsible for the sometimes observed fluctuations in postural control, proprioception or fluid retention during different phases of the menstrual cycle. Likewise, the observed fluctuations in injury risk might be affected by multiple variables. While the mechanical properties of the musculoskeletal system, balance and proprioception are likely candidates to be amongst those variables, other contributing factors might not have been identified or investigated yet. E.g. behavioural changes, which have been reported to be associated with certain phases of the menstrual cycle,^54^ might as well have an effect on the injury risk.

One limitation of the study is that it was not possible to differentiate between oral contraceptive users and non‐users, as we were not able to recruit enough non‐contraceptive users. This also means that the results of this study are predominantly applicable to athletes using a hormonal method of contraception. Also, hormone levels were not determined, and thus no correlations between hormone levels and the investigated parameters were analyzed. However, previous studies already failed to show these correlations as described above, so we refrained from investigating them. Furthermore, changes in the investigated variables still might have been occurred, being too small to be detected with the small sample size and by measuring only one menstrual cycle.

## 5 PERSPECTIVE

Our ice hockey players constant performances in the executed tests suggests that most healthy young women on hormonal contraception are unlikely to be affected by major fluctuations in proprioception, dynamic stability and fluid retention along the menstrual cycle. It appears that the effect of hormonal changes along the menstrual cycle, often given as an explanation why a study population is restricted to men, might have been overestimated in general.

In conclusion, regarding the parameters investigated in our study, one should be able to include healthy young women on hormonal contraception in a study population, without having to fear hormone associated fluctuations. We furthermore conclude that fluctuations in balance or proprioception within the menstrual cycle are either non‐existent or so small, that they should not have to be taken into account by athletes and their coaches when planning training or competition.

However, fluctuations might still occur in individual cases or in association with other factors, such as e.g. the premenstrual symptom. The challenge remains to identify the specific ones at risk to suffer from menstrual cycle associated performance impairment or injury. Simple cause and effect relations failed so far to provide a sufficient explanation for menstrual cycle phase associated injuries. Thus, future studies might have to apply complex analytic methods including multiple independent variables to uncover the factors responsible for fluctuations in injury rate and performance.

## DISCLOSURE STATEMENT

In accordance with ethical obligations as researcher, the authors report no conflicts of interest that may affect the research reported in the enclosed paper. We declare that the results of the study are presented clearly, honestly, and without fabrication, falsification, or inappropriate data manipulation.
